# Characterization of fosfomycin resistance and molecular epidemiology among carbapenem-resistant *Klebsiella pneumoniae* strains from two tertiary hospitals in China

**DOI:** 10.1186/s12866-021-02165-7

**Published:** 2021-04-11

**Authors:** Haichen Wang, Changhang Min, Jun Li, Ting Yu, Yongmei Hu, Qingya Dou, Mingxiang Zou

**Affiliations:** 1 Department of Clinical Laboratory, Xiangya Hospital, Central South University, No. 87, Xiangya Road, Kaifu District, Changsha, 410008 Hunan China; 2 Department of Infection Control Center, Xiangya Hospital, Central South University, No. 87, Xiangya Road, Kaifu District, Changsha, 410008 Hunan China

**Keywords:** Carbapenem resistance, *Klebsiella pneumoniae*, Fosfomycin, *fosA3*

## Abstract

**Background:**

Fosfomycin has been proven to be a vital choice to treat infection caused by multidrug resistance bacteria, especially carbapenem-resistant *Klebsiella pneumoniae* (CRKP). However, fosfomycin resistant cases has been reported gradually. In this study, we reported the fosfomycin-resistant rate in CRKP strains and further revealed the molecular mechanisms in resistance gene dissemination.

**Results:**

A total of 294 non-duplicated CRKP strains were collected. And 55 fosfomyin-resistant strains were detected, 94.5% of which were clustered to sequence type (ST) 11 by PCR followed up sequencing. PFGE further revealed two major groups and four singletons. The positive rates of genes responsible to fosfomycin and carbapenem resistance were 81.8% (*fosA3*), 12.7% *(fosA5*) and 94.5% (*bla*_KPC-2_), respectively. Genomic analysis confirmed insertion sequence (*IS*) 26 was the predominant structure surrounding *fosA3*. The *fosA3* genes in six isolates were located on plasmids which were able to transfer to *E. coli* J53 recipient cells by means of conjugation.

**Conclusions:**

Although the resistant rate of CRKP to fosfomycin is relatively low in our area, considering its gene is located on transferrable plasmid and inserted in IS structure, continuous monitoring is still needed.

**Supplementary Information:**

The online version contains supplementary material available at 10.1186/s12866-021-02165-7.

## Introduction

Carbapenem resistant *Klebsiella pneumoniae* (CRKP) has become a great threat to public health. The dissemination of CRKP causes severe morbidity and mortality, due to few antibiotics available for the treatment [1].

Fosfomycin is a bactericidal antibiotic which inhibits the biosynthesis of cell wall by irreversibly binding with UDP-N-acetylglucosenol acetonyl transferase (MurA), an essential enzyme for peptidoglycan biosynthesis. Fosfomycin is commonly used in uncomplicated urinary tract infection caused by susceptible organisms [2, 3]. In recent years, fosfomycin has been proven to be effective against multidrug-resistance bacteria and recommended as alternative option for treatment of CRKP [4].

During the medical application of fosfomycin, resistant strains has been continually reported [5]. Three resistance mechanisms to fosfomycin have been reported, including the fosfomycin modified enzymes, amino acid substitutions of the antibiotic MurA target and mutations of fosfomycin transport system (GlpT and UhpT) and its regulatory genes [5–7]. The resistance mechanisms exhibit divergences among different regions [5]. The fosfomycin modified enzymes is the predominant mechanism of fosfomycin resistance in China. More than ten *fos* genes have been identified [8, 9]. Gene *fosA3* is the most prevailing variant, mainly distributed in Asia, and can spread horizontally [10]. Therefore, the monitor of fosfomycin resistance is necessary to maintain fosfomycin effectiveness.

In the present study, we intended to investigate the in vitro antibacterial activity against CRKP from two teaching hospitals in China and further explore the resistance mechanism.

## Result

### Antibiotic susceptibility profiles

Among the 294 tested CRKP strains, 55 strains were resistant to fosfomycin (MIC ≥256 μg/mL). The fosfomycin resistant rates for two hospitals were 14.3 and 18.9%, respectively. All the fosfomycin resistance strains were highly resistant to tested antibiotics, including amikacin (AK), aztreonam (ATM), cefotaxime (CTX), cefotaxime (CRO) and ceftriaxone (FEP), with the minimum inhibitory concentration (MIC) at which 50% isolates were inhibited (MIC50) were greater than or equal to 256 μg/mL. All the strains were susceptible to polymyxin B (PB). The antibiotic susceptibility results were showed in Table 1.

**Table 1:** Antimicrobial susceptibility results of 55 CRKP clinical isolates

Antimicrobial agents	MIC (μg/mL)			number of isolates (%)		
	Range	MIC_50_	MIC_90_	S	I	R
CTX	256–> 256	> 256	> 256	0 (0.0)	0 (0.0)	55 (100)
CRO	256–> 256	> 256	> 256	0 (0.0)	0 (0.0)	55 (100)
FEP	128 – > 256	> 256	> 256	0 (0.0)	0 (0.0)	55 (100)
MEM	8–> 256	256	> 256	0 (0.0)	0 (0.0)	55 (100)
ATM	2–> 256	> 256	> 256	1 (1.8)	0 (0.0)	54 (98.2)
FOS	256–> 512	> 512	> 512	0 (0.0)	0 (0.0)	55 (100)
AK	1–> 256	> 256	> 256	4 (7.3)	0 (0.0)	51 (92.7)
TGC	1–8	2	8	22 (40.0)	8 (14.5)	25 (45.5)
PB	0.25–1	0.5	0.5	55 (100)	0 (0.0)	0 (0.0)

MIC_50_, minimum inhibitory concentration for 50% of the isolates; MIC_90_, minimum inhibitory concentration for 90% of the isolates

*S* susceptibility, *I* intermediate, *R* resistance

*CTX* cefotaxime, *CRO* ceftriaxone, *FEP* cefepime, *MEM* meropenem, *ATM* aztreonam, *FOS* fosfomycin, *AK* amikacin, *TGC* tigecycline, *PB* polymyxin B

The susceptibility profiles were analyzed according to the CLSI guidelines for CTX, CRO, FEP, MEM, ATM, FOS and AK, and EUCAST for PB and TGC

### Screening for carbapenem and fosfomycin resistance genes

For carbapenem-resistance genes, the detection rate of *bla*_KPC_ was 94.5% (52/55) in the CRKP isolates and all *bla*_KPC_ belonged to *bla*_KPC-2_. None *bla*_NDM_ and *bla*_OXA-48_ were identified in our study.

Among the 55 fosfomycin-resistant strains, 45 (81.8%) were positive with *fosA3* gene, 7 (12.7%) were *fosA5*, while none harbored with *fosA* or *fosC2* genes. For three strains which were negative for fosfomycin-resistance genes tested in our study, an amino acid substitution in Thr287Asn was discovered in fosfomycin target *murA*.

### Bacterial genotype

**Fig. 1:**
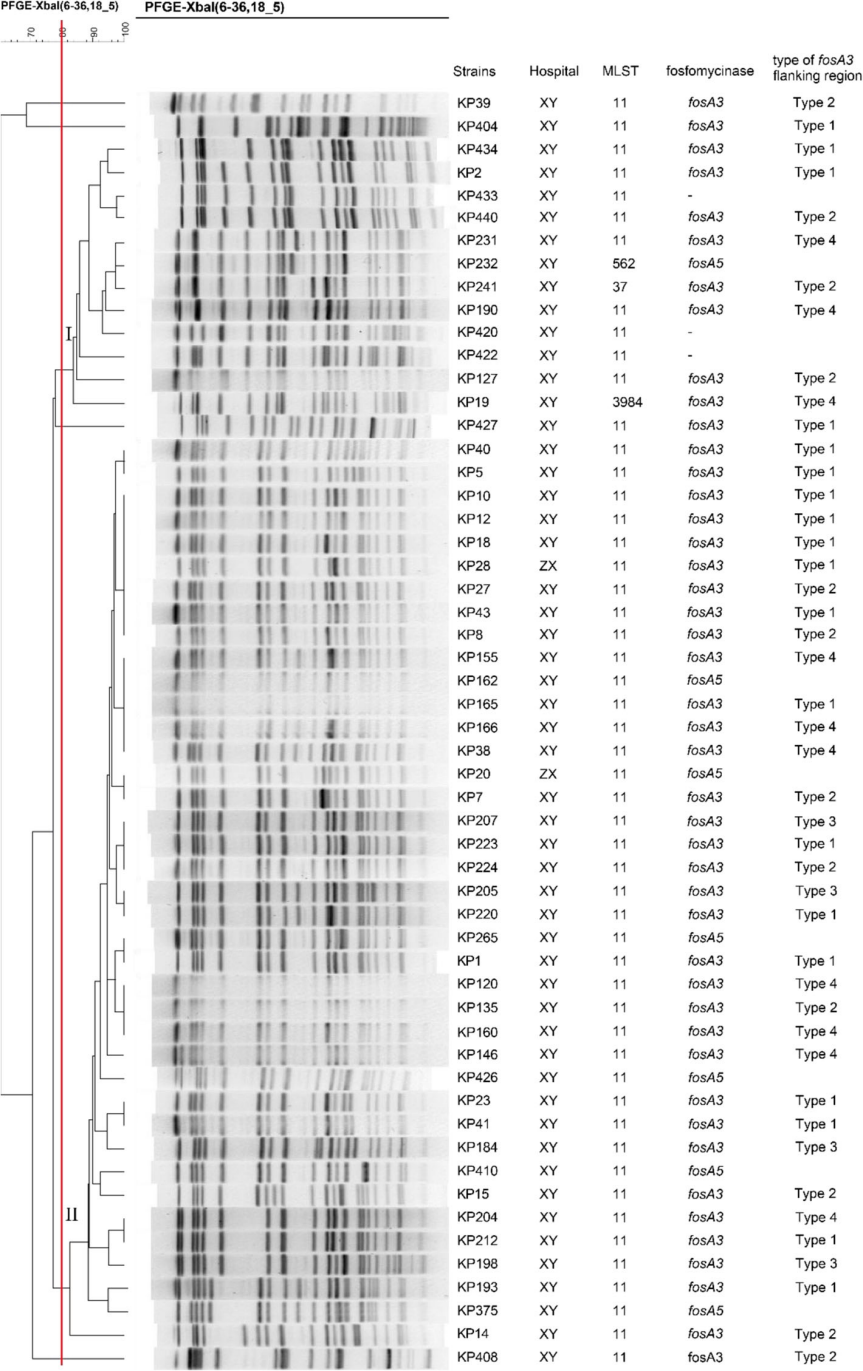
Dendrogram of relationships among 55 fosfomycin-resistant CRKP via the unweighted pair group method. Red line represents Dice coefficient equal to 80%

The dendrogram map conducted by pulsed-field gel electrophoresis (PFGE) revealed the genetic relationship between the fosfomycin-resistant strains. Two major groups (Group I and Group II) and four singletons were identified (Fig. 1). Group II was predominant that comprised 39 strains that were isolated in two hospitals.

In addition to multilocus sequence typing (MLST), 52 (94.5%) strains belonged to ST11. The rest of three strains belonged to ST 562, ST 37 and a new ST type, ST 3984, respectively.

### Genetic environment surrounding *fosA3* gene

**Fig. 2:**
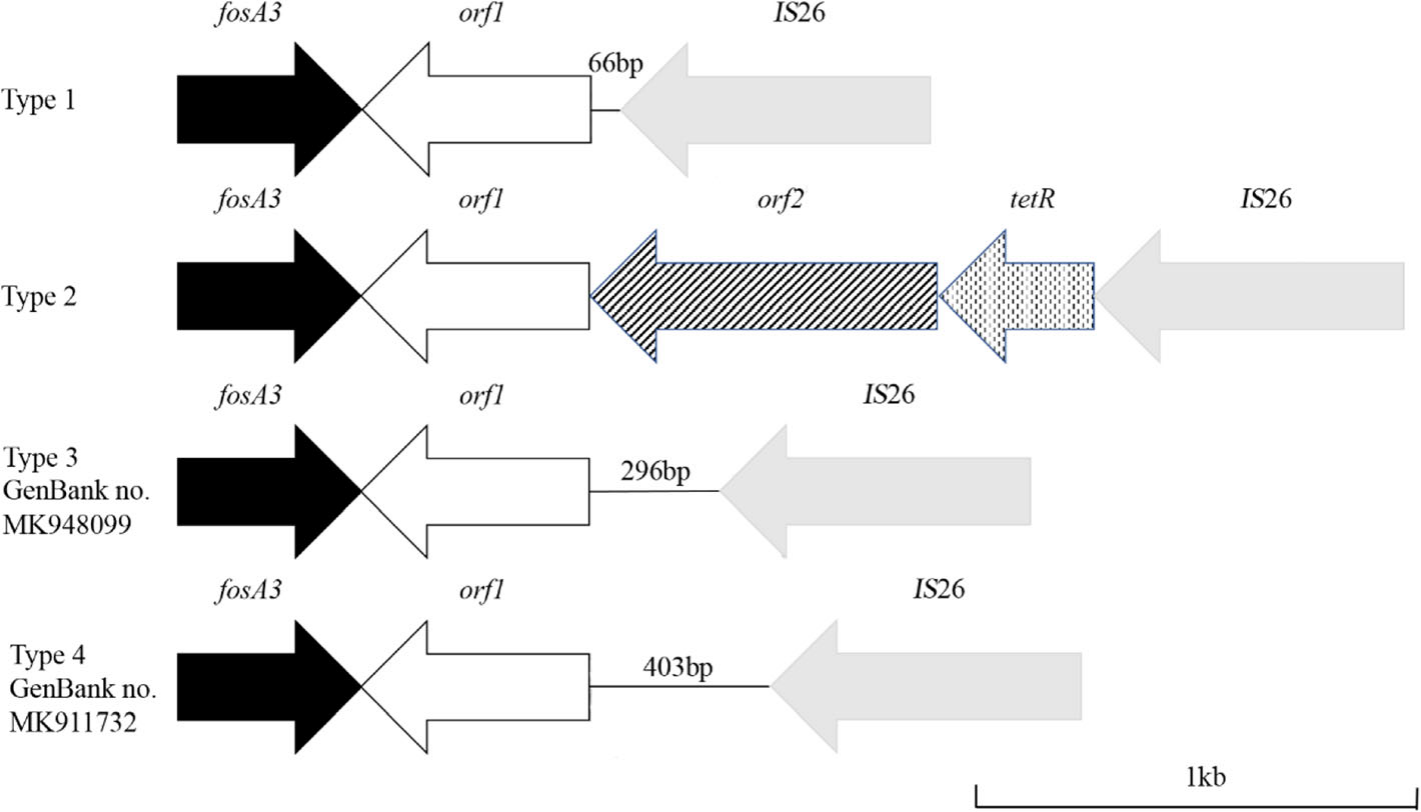
Schematic maps of four type of genetic environments between gene *fosA3* and downstream *IS*26

The genetic environment adjacent to *fosA3* was determined by PCR mapping. All the *fosA3* genes were located between two *IS*26 oriented in the opposite direction. The structure between *fosA3* and upstream *IS*26 was the same in all 45 strains. The length of intergenic region between upstream *IS*26/*fosA3* was 386 bp. However, four different downstream regions of *fosA3* were discovered and designated as type 1 to 4, with variable lengths between *fosA3* and the downstream *IS*26 (589, 819, 926 and 1811 bp). Type 1, accounting for 20 strains, consisted of *fosA3-orf1-IS*26 and shared 99.4% identity with the corresponding region of plasmid pKP 19–2029-KPC2 from *K. pneumoniae* strain KP19–2019 (GenBank no. CP047161). Twelve strains belonged to type 2, with a genetic background of *fosA3*-*orf1*-*orf2*-*tetR*-*IS*26, which was similar with that on plasmid p116753-KPC from *K. pneumoniae* strain 116,753 (GenBank no. MN891682). Two new types of *fosA3* downstream sequence were found in our study, namely type 3 and 4, accounting for four and nine strains, respectively, and registered as MK948099 and MK911732 in the GenBank. The schematic map for four types was shown in Fig. 2.

### Conjugation experiments and plasmid analysis

Among the 45 *fosA3* positive strains, 6 (13.3%) *fosA3* genes were transferable to *E. coli* J53 recipient. For the antibiotic susceptibility profiles, four transconjugants showed highly resistant to antibiotics tested, compared to *E. coli* J53 recipient. However, two transconjugants (TC5 and TC18) only showed an increase in the MIC value of fosfomycin and amikacin (Table 2). PCR confirmed the *bla*_KPC-2_ was absent in TC5 and TC18 (Figure S1).

**Table 2:** Antimicrobial susceptibility results of 6 *fosA3* isolates with capability of transconjugation and their transconjugants

Isolate	MIC (μg/mL)									plasmid type
	CTX	CRO	FEP	MEM	ATM	FOS	AK	TGC	PB	
KP5	> 256	> 256	> 256	> 256	> 256	> 512	> 256	4	0.5	IncN
KP18	> 256	> 256	> 256	> 256	> 256	> 512	> 256	4	0.5	IncN, IncF
KP165	> 256	> 256	> 256	256	> 256	> 512	> 256	4	0.5	ND
KP190	> 256	> 256	> 256	> 256	> 256	> 512	> 256	1	0.5	IncL/M
KP212	> 256	> 256	> 256	256	> 256	> 512	> 256	4	0.5	ND
KP223	> 256	> 256	> 256	256	> 256	> 512	> 256	4	0.5	ND
TC5	≤0.25	≤0.25	≤0.25	1	≤0.25	> 512	2	≤0.25	≤0.25	IncN
TC18	≤0.25	≤0.25	≤0.25	1	≤0.25	> 512	2	≤0.25	≤0.25	IncN
TC165	> 256	> 256	64	4	256	> 512	> 256	≤0.25	≤0.25	ND
TC190	8	> 256	8	2	128	> 512	> 256	≤0.25	≤0.25	IncL/M
TC212	> 256	> 256	32	4	128	> 512	> 256	≤0.25	≤0.25	ND
TC223	> 256	> 256	32	4	256	512	> 256	≤0.25	≤0.25	ND
EC J53	≤0.25	≤0.25	≤0.25	1	≤0.25	2	1	≤0.25	≤0.25	/

*KP K. pneumoniae*, *TC* transconjugant, *EC E. coli*, *ND* not detected

**Fig. 3:**
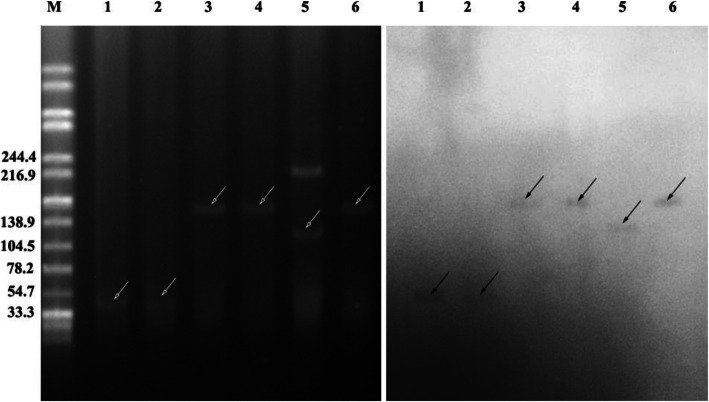
S1-PFGE and Southern blot hybridization of *fosA3* transconjugants. Bands with black arrows pointing showed the positive signals in Southern blot with *fosA3* probes. S1-PFGE was shown in left and Southern blot was shown in right. M = DNA ladder of *Salmonella serotype Braenderup* H9812 strain digested by XbaI. 1, *E. coli* J53 (KP5 plasmid); 2, *E. coli* J53 (KP 18 plasmid); 3, *E. coli* J53 (KP 165 plasmid); 4, *E. coli* J53 (KP 190 plasmid); 5, *E. coli* J53 (KP 212 plasmid); 6, *E. coli* J53 (KP 223 plasmid)

S1-PFGE, southern blotting and PCR-based replicon typing were used for plasmid analysis. S1-PFGE demonstrated that five in six transconjugants harbored single plasmid. The plasmids harbored in six transconjugants were assigned to the following incompatibility groups: IncN (*n* = 2), IncL/M (*n* = 1) and not determined (ND, *n* = 3) (Table 2). Southern blot analysis confirmed that the *fosA3* genes were located in the plasmids of different sizes (~ 40, 100 and 140 kb) in the transconjugants (Fig. 3).

For the rest 49 strains, the plasmids were assigned to the following incompatibility groups: IncF (*n* = 42) and ND (*n* = 3). Multiple replicons were detected in 4 strains (the data were shown in supplemental table).

## Discussion

Our study investigated the prevalence of fosfomycin resistance genes among 294 non-duplicate CRKP strains from two tertiary hospitals in two provinces. We have reported a resistance rate of 18.7%, indicating a relatively low resistant rate to fosfomycin, compared to a study conducted by Chen et al. (28.7%, 29/101 strains of CRKP) [11]. Probably because fosfomycin is not commonly used in the two settings. However, recently studies reported severe resistance rates to fosfomycin among CRKP in China between 2015 to 2020, ranging from 48.5 to 80% [12, 13]. The fast spread of fosfomycin resistance present further medical challenge for CRKP treatment, due to few antibiotics available. Another factor, which may impede the application of fosfomycin, is the difference between CLSI and EUCAST on MIC breakpoint. CLSI considers a MIC greater than or equal to 256 μg/mL to fosfomycin as resistance. However, EUCAST chooses 32 μg/mL as the breakpoint for fosfomycin to discriminate resistance. Furthermore, the CLSI breakpoint for fosfomycin only applies to *E. coli* urinary trait isolates. There is an urgent need for more clinical studies to determine the breakpoints for fosfomycin on *K. pneumoniae* systematic infection.

Polymerase chain reaction (PCR) screening revealed the plasmid gene *fosA3* was the predominant resistance mechanism. Gene *fosA3* was first discovered in a *E. coli* strain and transferred with resistance genes including CTX-M and *rmtB*, resulting in highly resistant to fosfomycin [14]. Previous studies have demonstrated that the plasmid carrying *fosA3* were classified into incompatibility group IncF II, IncN, IncI1 IncB/O or not determined [15]. IncF plasmids are heterogeneous with variable size and frequently carry more than one replicon and resistance genes, contributing the fitness of the host [16]. In our study, IncF plasmid was also the predominant replicon type in all 55 strains. Interestingly, all the IncF plasmids were unable transferred to *E. coli* J53 by means of conjugation. Only six *fosA3* genes were transferable to *E. coli* J53 recipient. However, two transconjugants showed no increase in MIC values of antibiotics tested in our study, except for fosfomycin and amikacin (Table 2). PCR and southern blot analysis confirmed the *fosA3* genes of the two transconjugants were located in a plasmid around 40 kb (Fig. 3) and the absence of *bla*_KPC-2_ (Figure S1). The genes *fosA3* and *bla*_KPC-2_ coexist on a plasmid and can spread together by means of plasmid transfer. Jiang et al. observed a plasmid co-harbored *fosA3* and *bla*_KPC-2_ on different transposon systems [17]. Li et al. reported a IncP1 plasmid co-harbored *fosA3* and *bla*_KPC-2_ in the same Tn*1721*-Tn*3*-like composite transposons [18]. The *rmtB* gene, which contributes to the resistance of aminoglycosides, is frequently located on plasmid with *fosA3*. So we also tested the *rmtB* gene among six transconjugants by PCR and confirmed that five transconjugants harbored *rmtB* gene (Figure S1). The coexistences of these resistance genes and the horizontal gene transfer may promote the spread of *fosA3* and fosfomycin resistance by co-selection, due to the excessive use of carbapenems and aminoglycosides for treatment of bacterial infection.

As for the 3 strains which were negative for fosfomycin resistance genes, an amino acid change on *murA* may account for the resistance, which was also reported in other study [19]. However, no change in active binding site of fosfomycin (Cys115 residue) and three conserved positively charged residues (Lys22, Arg120 and Arg397) in *murA* was discovered, so further study is needed to reveal the influence of Thr287Asn in *murA* on fosfomycin susceptibility.

Insertion sequence *IS*26 surrounds *fosA3* gene and plays an important role in the dissemination of *fosA3*. Different studies have reported a s *IS*26-*fosA3*-*IS*26-like structure, which was similar with the structures in our study, while the length between *IS*26 and *fosA3* was variable [5]. The sequences of MK948099 and MK911732 showed some differences with that of plasmids known in GenBank. The sequence of MK948099 showed a difference of 47 base pairs compared with corresponding fragment of plasmid pHNGD46 (GenBank no KJ668701.1) in *E. coli* GDC46.

Based on the PFGE pattern, we disclosed that clone dissemination may play an important role in the spread of fosfomycin resistance, which is consistent with different studies on CRKP [20]. More importantly, group II contained strains from Cangzhou Central Hospital (KP28) and Xiangya hospital (KP10, KP12 and KP18), which shared similar PFGE bands and carried gene *fosA3*. It could be a clue that the spread of *fosA3* among CRKP may attribute to clone expansion. So, it is urgent to monitor the fosfomycin resistance and use the antibiotics with caution to prevent further spread of fosfomycin resistance. According to MLST, ST11 was the predominant type in our study, which is in agreement with the fact the ST11 is primary sequence type in Asia for CRKP [21].

## Conclusion

The fosfomycin resistance rate of CRKP strains is low in our study. The main mechanism of fosfomycin resistance is plasmid-mediated genes, which located on transferrable plasmid and inserted in *IS* structure, so further monitoring the fosfomycin resistance should be strengthened.

## Materials and methods

### Bacteria source

A total of 294 non-duplicate CRKP strains were collected from two tertiary hospitals (Cangzhou Central hospital from Hebei province and Xiangya hospital from Hunan province) in China between December 2016 and March 2019. The sample sources included blood (*n* = 48), sputum (*n* = 132), urine (*n* = 34), abscess (*n* = 22) and other samples (*n* = 58). CRKP was defined as strains with MIC values ≥4 μg/ml for IPM or MEM based on Clinical and Laboratory Standards Institute 2018 (CLSI) guidelines.

### Bacteria identification and antimicrobial susceptibility test (AST)

The strains were identified by VITEK-2 Compact system (bioMérieux, Marcy L’Etoile, France) or Microflex™ MALDI-TOF MS system (Bruker Daltonik, Bremen, Germany).

Broth microdilution method with Mueller-Hinton broth (Oxoid, unipath, UK) was used for AST according to the CLSI 2018 guidelines [22]. Minimum inhibitory concentration (MIC) for fosfomycin was determined by agar dilution method with Mueller-Hinton agar supplemented with 25 μg/mL glucose-6-phosphate. The susceptibility profiles were analyzed according to the CLSI guidelines, and the European Committee on Antimicrobial Susceptibility Testing breakpoints (EUCAST, www.eucast.org) for polymyxin B and tigecycline.

### Detection on resistance mechanisms of carbapenem and fosfomycin

Further confirmation test on the resistance genes was completed by means of PCR. The carbapenem-resistance genes, including *bla*_KPC_, *bla*_NDM_ and *bla*_OXA-48_, and fosfomycin-resistance genes, such as *fosA*, *fosA3*, *fosA5* and *fosC2*, were involved in our study according to previous reports [23–25]. We also analyzed the variants of the *bla*_KPC_ genes by PCR and follow up sanger sequencing [26].

For strains which were negative for tested fosfomycin-resistance genes, *murA* gene were amplified according to previous work [19]. The products were sequenced and compared with the *murA* gene sequence of fosfomycin-sensitive *K. pneumoniae* K68 stains (GenBank no. KT334183) available at the National Center for Biotechnology Information website.

### Bacteria homology analysis

PFGE was employed to analyze the genomic background among Fosfomycin-resistant CRKP strains according to the standard protocol [27]. Briefly, the genomic DNA was digested with XbaI restriction enzyme for 12 h and separated by running PFGE electrophoresis with 1% agarose gel at 12 °C and 5.5 V/cm, with alternating pulses at a 120° angle in 0.5–70 s pulse time gradient for 21 hs. BioNumerics software (Applied Maths) was used for dendrogram analysis using the dice similarity coefficient. Strains were classified as the same PFGE group if they possessed ≥80% genetic similarity [28]. *Salmonella enterica* H9812 was used as the size marker.

MLST was used to analyze ST type of CRKP. Seven house-keeping genes of *K. pneumoniae* (*rpoB*, *gapA*, *mdh*, *pgi*, *phoE*, *infB* and *tonB*) were amplified and the products were sequenced. The ST type was analyzed according to protocol of Pasteur website (http://bigsdb.web.pasteur.fr).

### Conjugation experiment and plasmid typing

The conjugation experiments were used for the *fosA3* strains to examine the transferring capability of plasmids. The sodium azide-resistant *E. coli* J53 was used as recipient strain and filter-mating method was performed according to reported procedures with 64 μg/mL fosfomycin and 200 μg/mL sodium azide [29]. PCR and antibiotic susceptibility tests for the transconjugants were conducted to confirm the transferred *fosA3* and *bla*_KPC_ genes.

Plasmid DNA of all strains was extracted by E.Z.N.A. Endo-free Plasmid DNA Mini Kit (OMEGA, USA). The plasmid incompatibility group was identified by PCR-based replicon typing according to previous work, including HI1, HI2, I1/Ir, X, L/M, N, FIA, FIB, W, Y, R, FIC, A/C, T, FIIA, F and K [30].

### S1-PFGE and southern blotting

Southern blotting was employed to confirm the location of *fosA3* gene. Total genomic DNA was digested with S1 nuclease and electrophoresed with a CHEF-Mapper XA PFGE system (Bio-Rad, USA) for 16 h at 14 °C and 6 V/cm, with alternating pulses in 2.16–63.8 s pulse time. The DNA fragments were transferred to nylon membranes (Millipore, USA) and hybridized with digoxigenin-labelled *fosA3*-specific probe. An NBT/BCIP color detection kit (Roche Applied Sciences, Germany) was employed to detect the fragments [31].

### PCR mapping of the flanking region of *fosA3* gene

The genetic environment around *fosA3* gene was analyzed according to previous work [23]. The PCR products were sequenced and compared using the Basic Local Alignment Search Tool (http://blast.ncbi.nlm.nih.gov/Blast.cgi). Sequences reported here have uploaded to NCBI website with accession numbers of MK948099 and MK911732.

## Supplementary Information

**Additional file 1: Figure S1.** PCR analysis of genes *bla*_KPC-2_ (A) and *rmtB* (B) of *fosA3* transconjugants. M = Marker. 1, *E. coli* J53 (KP5 plasmid); 2, *E. coli* J53 (KP 18 plasmid); 3, *E. coli* J53 (KP 165 plasmid); 4, *E. coli* J53 (KP 190 plasmid); 5, *E. coli* J53 (KP 212 plasmid); 6, *E. coli* J53 (KP 223 plasmid).

**Additional file 2. Table S1.** Isolate name, source, hospital, antimicrobial susceptibility results, homology analysis and PCR results of all isolates that were analysed in this study.

## Data Availability

The datasets generated and analyzed during the present study are available from the corresponding author upon reasonable request.

## Abbreviations

CRKP: Carbapenem resistant *K. penumoniae*
AK: Amikacin
ATM: Aztreonam
CTX: Cefotaxime
CRO: Ceftriaxone
FEP: Cefepime
TGC: Tigecycline
PB: Polymyxin B
MEM: Meropenem
FOS: Fosfomycin
CLSI: Clinical and Laboratory Standards Institute
EUCAST: European Committee on Antimicrobial Susceptibility Testing breakpoints
MIC: Minimum inhibitory concentration
PCR: Polymerase chain reaction
PFGE: Pulsed-field gel electrophoresis
MLST: Multilocus sequence typing
AST: Antimicrobial susceptibility test
