# Supplementary material for: Characterization of fosfomycin resistance and molecular epidemiology among carbapenem-resistant Klebsiella pneumoniae strains from two tertiary hospitals in China
Source: BMC Microbiol. 2021 Apr 11;21:109. doi: 10.1186/s12866-021-02165-7 (PMC8037892; doi:10.1186/s12866-021-02165-7)
Supplement: Supplementary file 1 — Additional file 1: Figure S1. PCR analysis of genes blaKPC-2 (A) and rmtB (B) of fosA3 transconjugants. M = Marker. 1, E. coli J53 (KP5 plasmid); 2, E. coli J53 (KP 18 plasmid); 3, E. coli J53 (KP 165 plasmid); 4, E. coli J53 (KP 190 plasmid); 5, E. coli J53 (KP 212 plasmid); 6, E. coli J53 (KP 223 plasmid). [file 12866_2021_2165_MOESM1_ESM.docx]

**Supplemental data**

According to the susceptibility results, four transconjugants showed increased MIC value to amikacin. Furthermore, the *rmtB* gene are highly prevailing based on our previous work ^S1^. So we tested the *rmtB* gene in the six transconjugants. The PCR condition was as follows: initial denaturation at 94 °C for 5 min, followed by 30 cyclys at 94 °C for 30 s, at 54.5 °C for 30 s, and at 72 °C for 30 s, and a final extension at 72 °C for 5min ^S2^. The result showed that five transconjugants carried *rmtB*, therefore the resistance genes *fosA3*, *bla*_KPC_ and *rmtB* may be co-disseminated.





**Fig.S1** PCR analysis of genes *bla*_KPC-2_ (A) and *rmtB* (B) of *fosA3* transconjugants. M = Marker. 1, *E. coli* J53 (KP5 plasmid); 2, *E. coli* J53 (KP 18 plasmid); 3, *E. coli* J53 (KP 165 plasmid); 4, *E. coli* J53 (KP 190 plasmid); 5, *E. coli* J53 (KP 212 plasmid); 6, *E. coli* J53 (KP 223 plasmid).

Supplementary References

1. Li J, Zou MX, Wang HC, Dou QY, Hu YM, Yan Q, Liu WE. An outbreak of infections caused by a Klebsiella pneumoniae ST11 clone coproducing *Klebsiella pneumoniae* carbapenemase-2 and *rmtB* in a Chinese teaching hospital. Chinese medical journal. 2016; 129:2033-2039.
2. Bercot B, Poirel L, Nordmann P. Updated multiplex polymerase chain reaction for detection of 16S rRNA methylases: high prevalence among NDM-1 producers. Diagn Microbiol Infect Dis. 2011; 71(4):442-445.
